# Care Utilization and the Affordability of Care for Indian Health Service Beneficiaries

**DOI:** 10.1001/jamanetworkopen.2025.22045

**Published:** 2025-07-21

**Authors:** Matthew Tobey

**Affiliations:** 1 Department of Medicine, Massachusetts General Hospital, Boston

## Abstract

This cross-sectional study examines whether Indian Health Service benefits are associated with reduced cost-related barriers to care and increased care utilization among American Indian and Alaska Native individuals.

## Introduction

The Indian Health Service (IHS) provides health care at no cost to 1.7 million members of federally recognized tribes at 43 hospitals and 383 clinics located on or near American Indian and Alaska Native lands.^1^ Recent studies^2,3^ suggest that, among American Indian and Alaska Native individuals, access to IHS is associated with less cost-related avoidance of care, lower rates of medical debt, and greater prenatal care utilization.

To further explore financial and care utilization outcomes among American Indian and Alaska Native individuals, I analyzed data from the Medical Expenditure Panel Survey (MEPS) Household Component, a telephone interview survey of US households that records health care utilization, health benefits, and costs. I hypothesized that IHS benefits would be associated with reduced cost-related barriers to care and increased care utilization.

## Methods

I pooled data from 2018 to 2022 MEPS Household Component surveys and performed a cross-sectional analysis following MEPS pooling and subpopulation analysis guidance.^4^ This study adheres to STROBE reporting guidelines. The Massachusetts General Brigham institutional review board determined the study not to represent human participants research; thus, informed consent was not needed, in accordance with 45 CFR §46.

In MEPS Household Component telephone interviews, respondents provide detailed information regarding health-related finances and utilization, self-report race, and indicate IHS beneficiary status. A question regarding IHS benefits was first introduced in 2018. All members of federally recognized tribes are eligible for care at IHS; however, only those living within an accessible distance to an IHS site are likely to identify as beneficiaries. Comparing American Indian and Alaska Native IHS beneficiaries with nonbeneficiaries, I summarized demographic factors, poverty, insurance coverage, and self-reported health status, following MEPS variable definitions.^5^ For self-reported health status, which MEPS collects multiple times per year, I utilized the most recent nonmissing response following a common approach.^6^ With these variables and survey year as covariates, I performed a logistic regression for avoidance of care due to cost and Poisson regressions for the annual number of outpatient, emergency department, and inpatient visits, and reported adjusted values. In a sensitivity analysis, I analyzed 2020 to 2021 separately from the other years. Statistical significance was set at 2-sided *P* < .05. Data were analyzed with Stata statistical software version 18.0 (StataCorp).

## Results

Pooled 2018 to 2022 unweighted MEPS observations included 1149 American Indian and Alaska Native individuals, 297 of whom reported IHS benefits. Compared with the US population, American Indian and Alaska Native individuals lived less frequently in the Northeast, lived more frequently in the South, more frequently reported fair or poor health, less frequently had private insurance, and were more frequently uninsured (Table).

**Table.** Characteristics and Outcomes of American Indian and Alaska Native Individuals by Indian Health Service Beneficiary Status, MEPS Household Component, 2018-2022^a^

Characteristic	American Indian and Alaska Native individuals, weighted No. (%)		US population, adjusted No. (%)
	IHS beneficiaries	Not IHS beneficiaries	
Survey population, annual mean, No. (% of total population)			
Unweighted survey population	297 (0.22)	852 (0.62)	137 545
Weighted survey population	746 634 (0.23)	1 908 646 (0.58)	326 632 275
Characteristic, annual mean			
Age, mean (95% CI), y	37.6 (31.0-44.1)	36.6 (34.0-39.3)	39.3 (38.8-39.7)
Sex			
Female	320 489 (42.9)	426 146 (49.1)	167 608 806 (50.9)
Male	426 146 (57.1)	972 433 (51.0)	161 705 697 (49.1)
Census region			
Northeast	1954 (0.3)	169 323 (8.9)	55 704 299 (17.1)
Midwest	170 897 (22.9)	247 390 (13.0)	67 825 675 (20.8)
South	473 657 (63.4)	970 501 (50.9)	125 276 001 (38.4)
West	100 126 (13.4)	521 433 (27.3)	77 826 300 (23.8)
Poverty	58 182 (7.8)	336 254 (17.6)	38 293 966 (11.6)
Self-perceived health status			
Excellent	214 873 (28.9)	480 157 (25.2)	96 900 428 (29.5)
Very good	251 958 (33.8)	555 376 (29.1)	111 329 587 (33.9)
Good	168 016 (22.5)	599 369 (31.4)	86 528 195 (26.3)
Fair	87 120 (11.7)	215 933 (11.3)	26 564 496 (8.1)
Poor	24 667 (3.3)	57 810 (3.0)	7 263 585 (2.2)
Insurance coverage status			
Private plan	338 073 (45.3)	1 184 688 (62.1)	218 517 427 (66.4)
Public plan^b^	280 298 (37.5)	451 934 (23.7)	89 817 600 (27.3)
Uninsured	128 263 (17.2)	272 024 (14.3)	20 979 476 (6.)
Cost per utilization outcomes, annual mean^c^			
Care was delayed due to cost, % (95% CI)	3.8 (0.4-7.2)	9.7 (6.1-13.2)^d^	NA
Outpatient visits, No. (95% CI)	0.80 (0.31-1.29)	0.83 (0.49-1.16)	NA
Emergency department visits, No. (95% CI)	0.39 (0.27-0.51)	0.22 (0.16-0.29)^d^	NA
Inpatient discharges, No. (95% CI)	0.09 (0.05-0.14)	0.08 (0.05-0.11)	NA

Abbreviations: IHS, Indian Health Service; MEPS, Medical Expenditure Panel Survey; NA, not applicable.

^a^ All values reflect MEPS survey weighting and parameters, except for unweighted population.

^b^ Excludes IHS benefits, which are not considered health insurance because they do not guarantee financial coverage for external health services.

^c^ Adjusted in logistic regression (care delayed due to cost) or Poisson regression (annual outpatient visits, annual emergency department visits, annual inpatient discharges) for covariates age, sex, census region, poverty, self-perceived health status, insurance coverage, and MEPS Household Component survey year.

^d^ *P* < .05 for association between the outcome and IHS benefits in regression model.

In adjusted analysis, IHS benefits were associated with lower cost-related avoidance of care (3.8% [95% CI, 0.4%-7.2%] with benefits vs 9.7% [95% CI, 6.1%-13.2%] without; *P* = .02) and increased emergency department utilization (0.39 [95% CI, 0.27-0.51] visits per year vs 0.22 [95% CI, 0.16-0.29] visits per year; *P* = .01) (Table). In sensitivity analysis, differences in emergency department utilization were more pronounced in 2020 and 2021.

## Discussion

The results of this cross-sectional study agree with a prior study demonstrating that access to IHS is associated with lower cost-related avoidance of care.^2^ Although intuitive, it is nevertheless encouraging that no-cost care provided by IHS carries this association. This study also found that IHS benefits were associated with increased emergency department utilization, especially during the first years of the COVID-19 pandemic.

This analysis was limited by the number of observations of IHS beneficiaries. The MEPS Household Component does not oversample or explicitly weight American Indian and Alaska Native race. Also, MEPS reports only American Indian and Alaska Native sole race, a common limitation in health data, and results should be interpreted accordingly. Geographic data, apart from census region, are not reported by MEPS.

These findings underscore the role of IHS in reducing financial barriers to care for its beneficiaries. The results also highlight the contribution of IHS emergency departments to care access in American Indian and Alaska Native communities.

## Notes

Supplement.
Data Sharing Statement
