# Supplementary material for: Care Utilization and the Affordability of Care for Indian Health Service Beneficiaries
Source: JAMA Netw Open. 2025 Jul 21;8(7):e2522045. doi: 10.1001/jamanetworkopen.2025.22045 (PMC12281227; doi:10.1001/jamanetworkopen.2025.22045)
Supplement: Supplement. — Data Sharing Statement [file jamanetwopen-e2522045-s001.pdf]

Data Sharing Statement

Tobey. Care Utilization and the Affordability of Care for Indian Health Service Beneficiaries. *JAMA Netw Open*. Published July 21, 2025. doi:10.1001/jamanetworkopen.2025.22045

Data
Data available: Yes
Data types: Data (not involving human participants)
How to access data: MEPS website: https://www.meps.ahrq.gov/mepsweb/
When available: With publication

Supporting Documents
Document types: None

Additional Information
Who can access the data: NA
Types of analyses: NA
Mechanisms of data availability: public data: https://www.meps.ahrq.gov/mepsweb/
